# Supplementary material for: Overshoot during phenotypic switching of cancer cell populations
Source: Sci Rep. 2015 Oct 23;5:15464. doi: 10.1038/srep15464 (PMC4616026; doi:10.1038/srep15464)
Supplement: Supplementary Information [file srep15464-s1.pdf]

Supporting Information for "Overshoot in the phenotypic switching of cancer cell populations"

Alessandro L. Sellerio,[1,2] Emilio Ciusani,[3] Noa Bossel Ben-Moshe,[4] Stefania Coco,[5] Andrea Piccinini,[5] Christopher R. Myers,[6,7] James Sethna,[6] Costanza Giampietro,[8] Stefano Zapperi,[1,2,9,10] and Caterina A. M. La Porta[11,12]

[1]*Center for Complexity and Biosystems, Department of Physics, University of Milano, via Celoria 16, 20133 Milano, Italy*

[2]*CNR - Consiglio Nazionale delle Ricerche, Istituto per l'Energetica e le Interfasi, Via R. Cozzi 53, 20125 Milano, Italy*

[3]*Istituto Neurologico Carlo Besta, Milano, Italy*

[4]*Department of Physics of Complex Systems, Weizmann Institute of Science, Rehovot, Israel*

[5]*Dipartimento di Scienze Bomediche per la Salute, University of Milano, Milano, Italy*

[6]*Laboratory of Atomic and Solid State Physics, Physics Department, Cornell University, Ithaca, NY*

[7]*Institute of Biotechnology, Cornell University, Ithaca, NY*

[8]*Department of Bioscience, University of Milano, via Celoria 26, 20133 Milano, Italy*

[9]*ISI Foundation, Via Alassio 11C, Torino, Italy*

[10]*Department of Applied Physics, Aalto University, P.O. Box 14100, FIN-00076, Aalto, Finland*

[11]*Center for Complexity and Biosystems, Department of Bioscience, University of Milano, via Celoria 26, 20133 Milano, Italy*

[12]Corresponding author: caterina.laporta@unimi.it

Supplemental figure captions

Figure S1. CXCR6 negative cells are sorted and immediately re-analyzed for the same marker. The cells are stained with FITC anti-human CXCR6 (R& D System, Minneapolis, MN) and flow cytometry analysis is performed using a FACSAria flow cytometer (Becton, Dickinson and Company, BD, Mountain View, CA). Data are analyzed using FlowJo software (Tree Star, Inc., San Carlos, CA). For each flow cytometry evaluation, a minimum of 5×10^5 cells are stained and at least 50000 events are collected and analyzed. Non-specific mouse IgG is used as isotype control.

Figure S2. ABCG2 negative cells are sorted from IgR39 cells and plated under standard growth condition. After 3, 10 and 20 days,the cells are collected and analyzed by flow cytometry. Non-specific mouse IgG used is as isotype control. Unsorted cells are reported (U). Flow cytometry analysis is performed using a FACSAria flow cytometer (Becton, Dickinson and Company, BD, Mountain View, CA). Data are analyzed using FlowJo software (Tree Star, Inc., San Carlos, CA). For each flow cytometry evaluation, a minimum of 5×10^5 cells are stained and at least 50000 events are collected and analyzed.

Figure S3. CXCR6-CD271 double positive cells in human melanoma IgR39 cells. Subconfluent cells are incubated with both FITC anti-human CXCR6 (R& D System, Minneapolis, MN) and PE anti human CD271 (BD Pharmingen) and analyzed using flow cytometry. Non-specific mouse IgG used is used as isotype controls. Flow cytometry analysis is performed using a FACSAria flow cytometer (Becton, Dickinson and Company, BD, Mountain View, CA). Data are analyzed using FlowJo software (Tree Star, Inc., San Carlos, CA). For each flow cytometry evaluation, a minimum of 5×10^5 cells are stained and at least 50000 events are collected and analyzed.

Figure S4. STRs analyses of unsorted (WT), CXCR6 positive and negative IgR39 cells using NGMSelect kit (Applied Biosystems).

Figure S5. STRs analysis of unsorted (WT), CXCR6 positive and negative IgR39 using and Powerplex 16 kit (Promega).

Figure S6. a) Typical example of electropherogram of one of the amplified libraries.

Bioanalyzer 2100 to verify the presence of the peaks corresponding to the microRNAs (peaks range from 145 bp to 158 bp). b) Final library sequenced.Bioanalyzer 2100 to verify the presence of the peaks corresponding to the microRNAs (peaks range from 145 bp to 158 bp).

Figure S7. Identification of differentially expressed pathways at the overshoot. a) The color map shows the number of miRNAs targeting each pathway, according to the Diana-Mir-Path database, for the miRNA that are differentially expressed in CXCR6 and CD271 negative cells at different times. b) The number of genes targeted in each pathways for the same condition as in a).

Figure S8. Expression of regulatory factor for miRNA–222 silenced cells. a) The fold change of the expression level of regulatory factors in CXCR6 negative cells three days after sorting as compared with unsorted cells is shown for miRNA-222 silenced cells (left column) and wild type cells (right column, data already reported in Fig. 5). b) As control, we show the fold change in the expression level of regulatory factors in miRNA-222 and gapdh silenced unsorted cells as compared with wild type unsorted cells.

Figure S9. Detection of BRAF 1796T3A (V599E) variant. Heterozygous for the mutant allele 1796T3A is found by sequencing analysis (arrow).

Supplemental figures

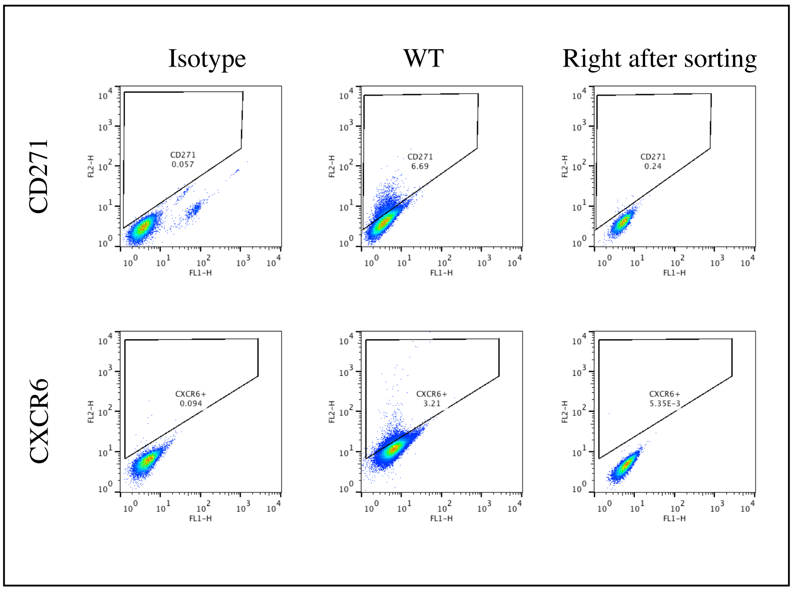

Figure S1:

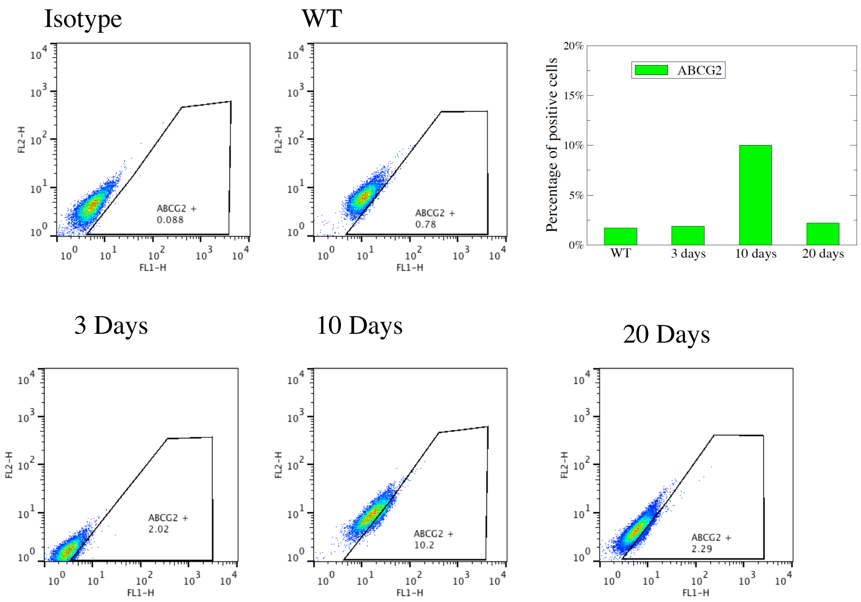

Figure S2:

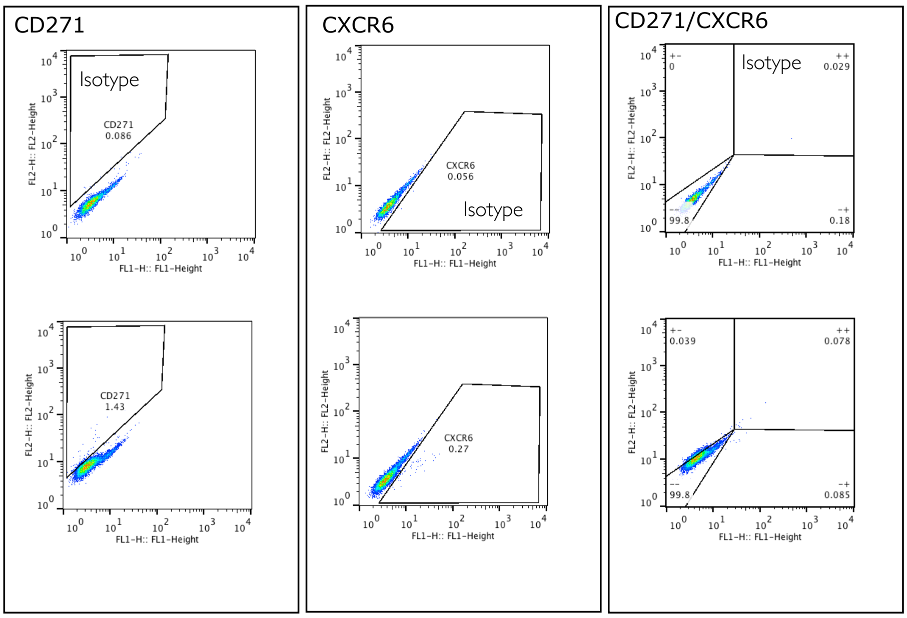

Figure S3:

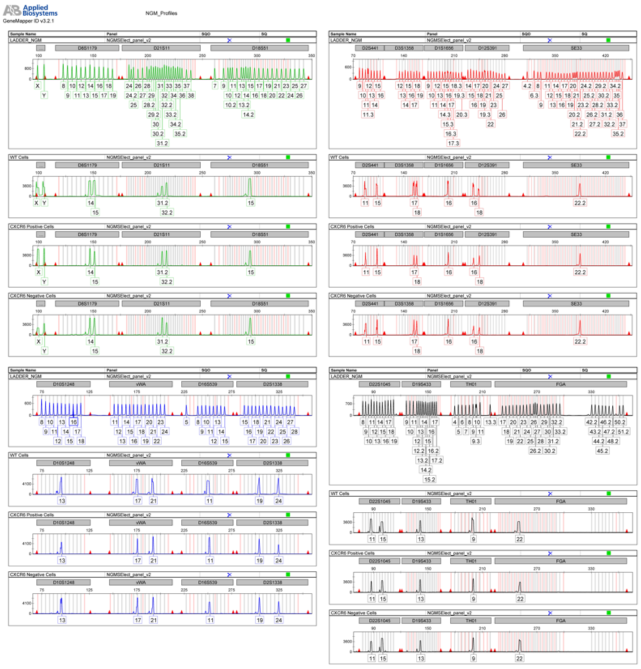

Figure S4:

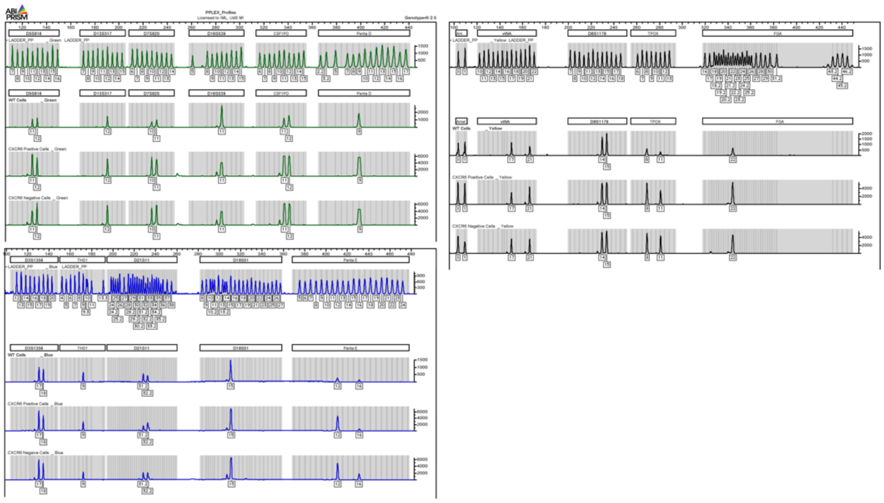

Figure S5:

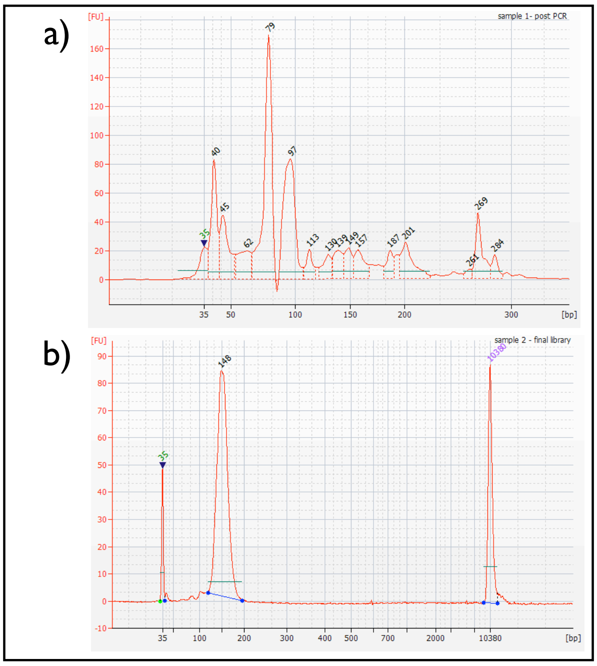

Figure S6:

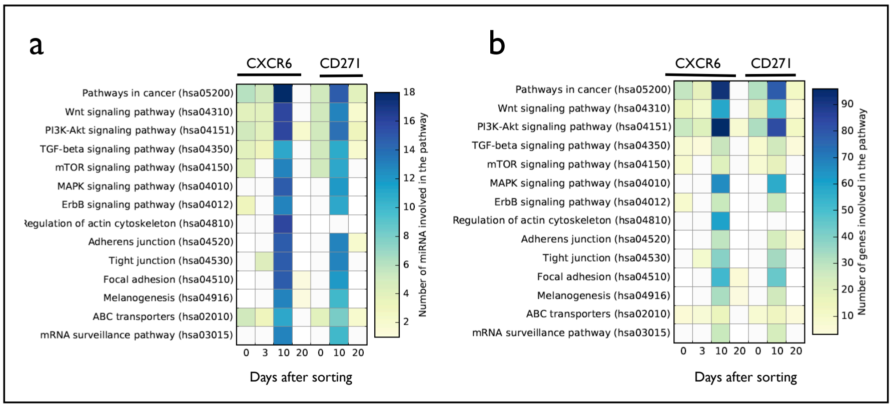

Figure S7:

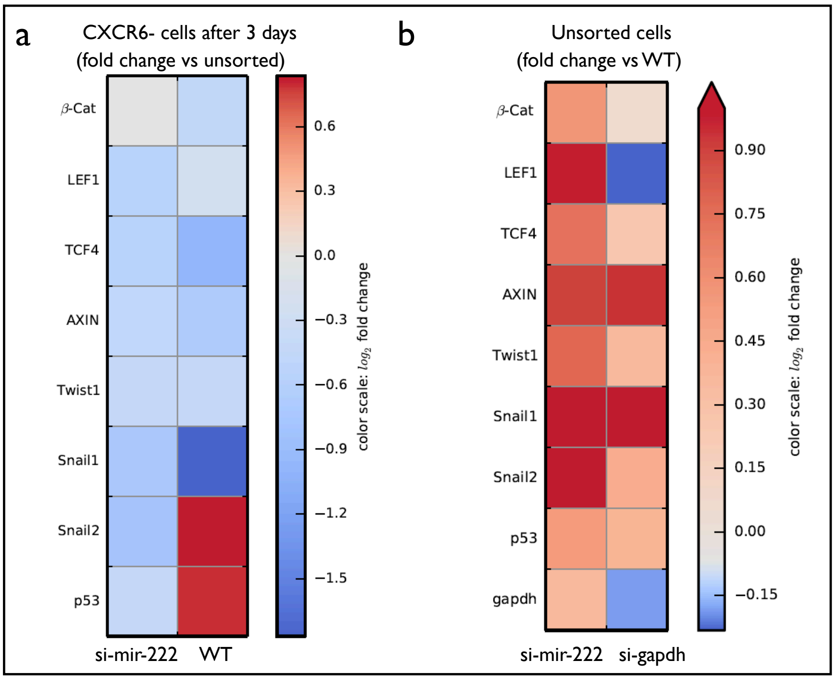

Figure S8:

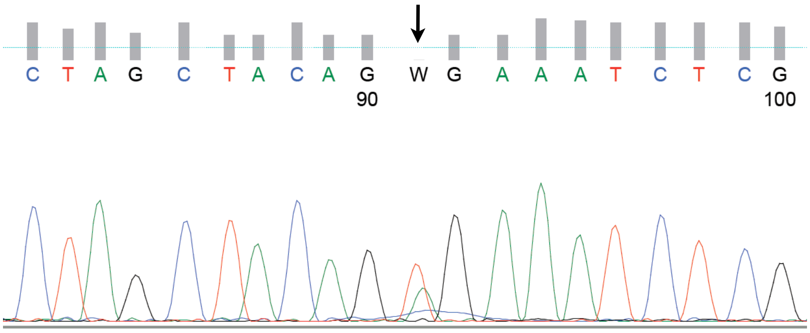

Figure S9:

Supplemental tables

STR Locus	Chromosomal Location
D10S1248	10q26.3
vWA	12p13.31
D16S539	16q24.1
D2S1338	2q35
Amelogenin X:	p22.1-22.3
Y:	p11.2
D8S1179	8q24.13
D21S11	21q11.2-q21
D18S51	18q21.33
D22S1045	22q12.3
D19S433	19q12
TH01	11p15.5
FGA	4q28
D2S441	2p14
D3S1358	3p21.31
D1S1656	1q42.2
D12S391	12p13.2
SE33	6

Table S1: NGM kit, technical details

STR Locus Location	Chromosomal defintion
Penta E	15q
D18S51	18q21.3
D21S11	21q1121q21
TH01	11p15.5
D3S1358	3p
FGA	4q28
TPOX	2p242pter
D8S1179	8q24.13
vWA	12p13.31
Amelogenin2	Xp22.122.3
Penta D	21q
CSF1PO	5q33.334
D16S539	16q24.1
D7S820	7q11.2122
D13S317	13q22q31
D5S818	5q23.332

Table S2: Powerplex 16 technical details

Gene	Forward	Reverse
CXCR6	ATGGCAATGTCTTTAATCTCGACAA	TGAAAGCTGGTCATGGCATAGTATT
PTEN	GAGGGATAAAACACCATG	AGGGGTAGGATGTGAACCAGTA
SOX2	AAGAGAACACCAATCCCATCCA	AGTCCCCCAAAAAGAAGTCCA
OCT4	AGTGAGAGGCAACCTGGAGA	ACACTCGGACCACATCCTTC
Nanog	GATTTGTGGGCCTGAAGAAA	AAGTGGGTTGTTTGCCTTTG

Table S3: Primers for Real Time PCR

Supplemental Data:

The list of miRNA expressed by unsorted IgR39 cells for two independent experiments.
